# Lead Exposures from Car Batteries—A Global Problem

**DOI:** 10.1289/ehp.0901163

**Published:** 2009-12

**Authors:** Richard Fuller

**Affiliations:** Blacksmith Institute, New York, New York, E-mail: fuller@blacksmithinstitute.org

In “Mass Lead Intoxication from Informal Used Lead Acid Battery Recycling in Dakar, Senegal,” Haefliger et al. (2009) described a problem throughout the developing world that is both tragic and only now beginning to be understood with respect to its extent and effect.

Eighteen children (and more since) died from acute lead poisoning in late 2008 in Dakar. These poisonings occurred because the individuals recycling car batteries melted slag without appropriate controls and without having any understanding of the toxicity of lead. Most of these recyclers were women who brought their children to their work sites without knowing the risks.

These problems are not restricted to Senegal. Without much effort, investigators from Blacksmith Institute have identified another 22 sites worldwide that are similar to this one. The identified sites are in cities in poor countries, especially in the tropics (e.g., the Dominican Republic, Philippines, Panama, El Salvador, Guatemala, India, Ghana, Jamaica) (Blacksmith Institute 2009).

Epidemiologic studies of exposed populations, such as the one in Senegal reported by Haefliger et al. (2009), are urgently needed to characterize exposures and identify related health effects. An earlier example of such a study was conducted in the Dominican Republic at Haina (also known as Bajos de Haina), which has been called the “Dominican Chernobyl.” This community is near an abandoned lead-acid battery recycling smelter, and most of the residents showed signs of lead poisoning.

The Haina site, as well as the surrounding area, was the scene of severe lead poisoning in the 1990s. In March 1997, 116 children were surveyed, and 146 children were surveyed in August 1997. Mean blood lead concentrations were 71 μg/dL (range, 9–234 μg/dL) in March and 32 μg/dL (range, 6–130 μg/dL) in August (Kaul et al. 1999). The study revealed that at least 28% of the children required immediate treatment and 5% had lead levels > 79 μg/dL, putting them at risk for severe neurologic sequelae at the time of the study. In the United States, the action level for blood lead concentration is 10 μg/dL (Centers for Disease Control and Prevention 2007; U.S. Environmental Protection Agency 2000).

The scientific findings from Haina (Kaul et al. 1999) drove a collaborative cleanup of this site, which has recently been completed. The Blacksmith Institute helped locate funding, worked closely with local authorities, and provided technical assistance to assure the cleanup was adequate. We are currently beginning a similar cleanup project in Dakar, at the site studied by Haefliger et al. (2009).

Almost all large urban centers in the developing world have a problem with recycling used lead acid batteries, and hundreds of thousands, if not millions, of children are exposed to lead from battery recycling. In humid conditions, car batteries need to be replaced every 2 or 3 years, and car use is increasing throughout the world, which will result in even more used batteries. Thus, this problem deserves our immediate and serious attention.
